# Community voices: sowing, germinating, flourishing as strategies to support inclusion in STEM

**DOI:** 10.1038/s41467-022-30981-6

**Published:** 2022-06-09

**Authors:** Luisa Maria Diele-Viegas, Thamara Santos de Almeida, Iris Amati-Martins, Christine D. Bacon, Cibele Cassia-Silva, Rosane G. Collevatti, Jéssica Fenker, Tabata Elise Ferreira Cordeiro, Giuliana Caldeira Pires Ferrari, Ana Clara Sampaio Franco, Luiza Flores Gasparetto, Juliana Hipólito, Camila Hohlenwerger, Beatriz Hörmanseder, Priscila Barreto de Jesus, Suzana dos Santos Matos, Daniela Pareja-Mejía, Beatriz Moraes Murer, Carla Brunner Pavone, Flávia B. Pilecco, Caren Queiroz-Souza, Alice Reis, Pamela Cristina Santana, Fernanda Dias-Silva, Lucy Souza, Mariana P. C. Telles, Jemilli Viaggi, Flávia Virginio

**Affiliations:** 1 Kunhã Asé Network of Women in Science, Salvador, BA Brazil; 2 Women in Zoology Network, Brasília, DF Brazil; 3 Biological Diversity and Tropical Conservation Graduate Program, Institute of Health and Biological Sciences, Federal University of Alagoas, Maceió, AL Brazil; 4 Graduate Program of Animal Biology, Federal University of Rio Grande do Sul, Porto Alegre, RS Brazil; 5 Oswaldo Cruz Foundation, Biodiversity Wildlife Health Institutional Platform (PIBSS/Fiocruz), Rio de Janeiro, RJ Brazil; 6 Landscape Ecology and Conservation Lab, Department of Ecology, Institute of Bioscience, University of São Paulo, São Paulo, SP Brazil; 7 Department of Biological and Environmental Sciences, University of Gothenburg, Gothenburg, Sweden; 8 Department of Plant Biology, Institute of Biology, University of Campinas, Campinas, SP Brazil; 9 Genetics and Biodiversity Lab, Institute of Biological Sciences, Federal University of Goiás, Goiânia, GO Brazil; 10 Australian National University, Canberra, Australia; 11 State Department of Education, Águas Lindas de Goiás, GO Brazil; 12 School of Criminology, Faculty of Law, University of Porto, Porto, Portugal; 13 Graduate Program of Neotropical Biodiversity, Federal University of the State of Rio de Janeiro, Rio de Janeiro, RJ Brazil; 14 Graduate Program of Ecology, Federal University Rio Grande do Sul, Porto Alegre, RS Brazil; 15 Graduate Program in Ecology Theory, Application and Values, Institute of Biology, Federal University of Bahia, Salvador, BA Brazil; 16 Institute of Biology, Federal University of Bahia, Salvador, BA Brazil; 17 Federal University of Espírito Santo, Vitória, ES Brazil; 18 Center of Natural and Human Sciences, Federal University of ABC, São Bernardo do Campo, SP Brazil; 19 Municipal Department of Education Rio de Janeiro, Rio de Janeiro, RJ Brazil; 20 Municipal Department of Education of Maricá, Maricá, RJ Brazil; 21 State University of Santa Cruz, Ilhéus, BA Brazil; 22 Science and Conservation Research Group, Ecology Department, Institute of Biosciences, São Paulo University, São Paulo, SP Brazil; 23 Laboratory of Ecology and Coastal Ecossystems, Marine Biology Center, Institute of Biosciences, São Paulo University, São Paulo, SP Brazil; 24 Department of Preventive and Social Medicine, Federal University of Minas Gerais, Belo Horizonte, MG Brazil; 25 Graduate Program in Ecology, Ecology Department, Biology Institute, São Paulo University, São Paulo, SP Brazil; 26 National Museum, Department of Vertebrates, Federal University of Rio de Janeiro, Rio de Janeiro, RJ Brazil; 27 Faculdade Estácio do Amazonas, Manaus, AM Brazil; 28 Graduate Program of Zoology, Federal University of Amazonas, Manaus, AM Brazil; 29 Graduate Program of Genetics, Conservation and Evolution Biology, National Institute of Amazonian Research, Manaus, AM Brazil; 30 ECMV, Pontifical Catholic University of Goiás, Goiânia, GO Brazil; 31 Ecology Department, Federal University of Rio de Janeiro, Rio de Janeiro, RJ Brazil; 32 Liga das mulheres pelos oceanos, Rio de Janeiro, RJ Brazil; 33 Research Group in Medical Entomology, Laboratory of Zoological Collections, Instituto Butantan, São Paulo, SP Brazil

**Keywords:** Scientific community, Ethics

## Abstract

Understanding gaps in academic representation while considering the intersectionality concept is paramount to promoting real progress towards a more inclusive STEM. Here we discuss ways in which STEM careers can be sown and germinated so that inclusivity can flourish.

The systems of oppression in society and science are deeply intertwined^1^, which becomes evident when analysing the senior positions at universities and scientific institutions and the editorial boards of scientific journals. Although it is possible to observe diversity among students and early career scientists, white, cisgender males from developed countries are still predominant in leadership positions^1^. The current academic gatekeeping system allows the maintenance of a discriminatory pyramid^1^ that excludes underrepresented groups along with their academic career in a phenomenon called the “leaky pipeline”^2^. Gender disparity (i.e., the disproportionate access to resources and participation in different environments between men and women) arises as a consequence of this pyramid^1^ and is potentialized by other systems of oppression, including racism, ableism, xenophobia, and 2SLGBTQIA + phobia (i.e., the prejudice against Two-Spirit, Lesbians, Gays, Bisexuals, Transexuals, Queer, Intersexual, Asexual and others)^3^. Despite being recognized in the literature, these different forms of discrimination in Science, Technology, Engineering, and Mathematics (STEM) are often approached separately^3^.

Individuals holding multiple marginalized identities present unique experiences that cannot be addressed when only singular identities are considered in research design^4^. By not focusing on intersecting identities, studies on diversity and inclusion lack critical information shaping the group outcomes, thus preventing the advance of a realistic and integrated discussion on the topic and the proposition of more concrete actions leading to a genuinely equal scenario. Intersectionality is the framework that discusses the simultaneous interaction between different systems of oppression operating to legitimize existing power relations^5^. While its theoretical principles are well known, the tools for putting it into practice are still emerging^4^. Thus, we base our discussion on a conceptual framework of Crenshaw’s intersectionality to focus on the experiences between and within groups across social identities^4,5^.

Being BIPOC (Black, Indigenous, and People of Colour)^6^ makes a person vulnerable to discrimination from birth. Colonization subjected the world to an ethnocentric perspective, producing a historical tradition of political and cultural domination^7^, and is still present to this day. The dominance of the Global North and its easy access to science resources undermines the Global South, imposing a hegemonic and colonizing way of doing science and generating a geopolitical and ethnic layer that segregates groups of people including native people, Latinos, and Africans^7^.

Similarly, the way in which society treats people with disabilities create barriers that may impact opportunities to participate in STEM^8^. Depending on their disability status and severity, people with disabilities may face different obstacles, including gaps in support services^8^. When gender identity is added to the equation, women with disabilities are more likely to be discriminated against than men^8^.

For people who are not cisgender (i.e., a person whose gender identity does not correspond to that traditionally associated with the anatomical sex at birth; not necessarily following a binary gender system^9^) or non-heterosexuals, discrimination begins when society identifies this dissociation from what is accepted as ‘normal’. This misconception of ‘normal’ will directly influence how this person is perceived by society and how STEM fields welcome this person.

Science is fed back by society’s patriarchal, white, and cis-heteronormative sovereignty and ableism domination^1^. Therefore, the hierarchy shaping society throughout centuries has also designed an oppressive and exclusionary scientific system. Consequently, it is essential to incorporate an intersectional approach focusing on the dialogue between social and political agendas to understand the nuances of social exclusion within academia, promote equality, diversity, and inclusion^5,7^, and truly address this multilayered issue^1,6,9^. Using the metaphor of plant development, we discuss the influences of the different systems of oppression on the career development of women scientists under an intersectional approach, highlighting possible paths to break these systems and promote the recruitment and retention of underrepresented groups in STEM fields.

## Sowing science: encouraging girls’ interest in STEM in early education

Even before the academic career starts, the cultivation of divergent interests in girls and boys crystallizes into different career choices among adults. Gender roles are imposed through toys in early childhood years, when girls receive toys associated with domestic activities, while boys are gifted toys exploring multiple skills, such as curiosity and exploration^10^. This divergence in stimuli available to children also influences gender stereotypes about intellectual abilities, with girls being less likely to believe that they are smart^10^. Moreover, scientists’ representation in cartoons, movies, and characters are mostly white and male, reinforcing this stereotype of scientists and influencing the idea of how a scientist should look, thus hampering the self-recognition of girls as future scientists^10^.

Science is taught from a cisnormative perspective in school, considering sex and gender as synonymous and binary^9,11^. This cisgender epistemology leads to a structural denial of “non-standard” genders that erase or diminish transgender experiences^11^, leading to high school dropout rates of transgender people^12^. Inequitable educational experiences also impact Black girls, who experience pervasive racial stereotypes and may be framed as disruptive and aggressive, being victims of abusive and punitive school discipline practices^13^. Girls with disabilities, in turn, face inequitable access to learning content in the classroom since the education system may provide little or no training on inclusive practices for educators, besides lacking the adequate physical infrastructure to support disabilities^8^. In addition, girls who experience adolescent pregnancy can drop out temporarily or permanently from studies, impacting their academic trajectory^13^. Such harsh environments are intensified in high-poverty communities, threatening positive youth development and these girls’ educational trajectories^13^.

Societal and family expectations can also directly influence a girl’s career choices and personal interests^12^. In many developing countries, scientists may lack employment benefits, job security, and financial stability^14^. Therefore, societal and family expectations may discourage girls from potential careers in STEM due to concerns about their future financial stability or social acceptance^14^. Although this discouragement can also occur for boys, girls’ social pressure may be more problematic if STEM is still considered a male field in their environment^15^.

We believe it is important to encourage girls to play with toys related to research, exploration, and logical and critical thinking. Some projects have focused on the development of STEM skills in girls, such as the *“Semeando Ciência”* promoted by Kunhã Asé Network of Women in Science (RKA; Sowing Science^14^) in Brazil and “For Girls in Science” promoted by the L’Oreal Foundation (https://www.forwomeninscience.com/authority/for-girls-in-science). Moreover, we need to stimulate girls’ sense of belonging^16^ by balancing the representation of scientists in movies and cartoons. Projects such as the “Skype a Scientist” (https://www.skypeascientist.com/) may be an excellent tool to connect students and scientists if focusing on a more inclusive representation. At the educational level, schools must provide a non-binary and inclusive education, repressing harassment and bullying to decrease the drop-off rates of underrepresented groups. Children must be taught to value diversity, while educators must be trained on inclusive practices. Finally, building self-confidence and nurturing the personal interests of girls and underrepresented groups from the primary school level is essential to empower these girls to choose their careers free of stereotypes and prejudices.

## Germinating scientists: supporting girls and women at early career stages

Even if girls overcome such initial barriers and proceed into STEM fields, they often experience inequality and sexism in academia^1^. Women face unbalanced opportunities for scientific collaborations since studies have shown that white male researchers, the most common in high positions, tend to collaborate and publish with peers^17,18^. This segregation process, known as homophily, excludes women and their intersections from informal networks, likely harming their career progress^18^. In addition, assumptions around knowledge and explaining without the appreciation of a woman’s expertise (often referred to as “mansplaining”), interruptions when speaking, misappropriating ideas, and underestimating their intelligence are some of many examples of the existing academic sexism^19^. Furthermore, harassment is a significant issue in all stages of a women’s academic career^1^, which may be more likely to occur if they are transgender, BIPOC, or people with disabilities^20^.

The cumulative effect of harassment and gender segregation in STEM may impact women’s mental health, stimulating issues like impostor and burnout syndromes, often leading to anxiety and depression^21^. One first step to fighting these issues is creating committees responsible for stimulating the community education concerning our implicit bias and promoting an academic environment where harassment is not tolerated. Institutions must promote a safe environment for women to report their harassers without reprisal and effectively punish harassers. To these committees to be effective, they must be diversified, composed not only by white cisgender men but also by representatives of underrepresented groups.

As mentioned before, the sense of belonging is also essential for retaining women and their intersections in the academic career^16^. STEM-identity, i.e., the internalized idea that one belongs to the STEM field they have chosen to follow^22^, may reduce the consequences of stereotype threat and psychosocial issues^23^. This identity can be stimulated during the different career stages through mentorship. Thus, mentors play a crucial role in retaining women and underrepresented groups in STEM, acting as career and institutional supporters through friendship, acceptance, counselling, and role modelling^24^. In addition, principal investigators play an important role in promoting equality during recruitment, networking, and paper collaboration^17^. Principal Investigators (PIs) have the leadership and power positions to create opportunities to include and stimulate the participation of underrepresented groups in science.

## Flourishing role models

The glass ceiling effect to which women are subjected is an invisible barrier hindering their access to positions of leadership and power since it generates hierarchical segregation and reinforces disparities in the academic environment^25^. Women may enter early career stages at greater numbers, but end up being replaced at higher levels in the academic chain mainly by white, cisgender men that manage to reach leadership and prestige positions^1^.

In many cases, women are still the main household members responsible for home and child care work—societal roles that have been imposed on women by a patriarchal culture for centuries^1,26^. Evidence suggests that married women with Ph.D. degrees are 11% less likely to continue a scientific career than women that are not married^26^. In contrast, married men with a Ph.D. degree are 12% more likely to continue an academic career than their single peers that are not married^26^. When women persist in their academic careers, the glass ceiling effect often may force them to rely on non-tenure positions, such as lab technicians and postdocs^27^. While lab technicians are less likely to be co-authors in high-profile papers than postdocs, postdocs usually may accumulate several additional roles in the laboratory, often without due recognition or without culminating in scientific publications^28^. These additional roles may include activities commonly related to a maternal perspective, such as administrative obligations, early career scientist training, and teaching^28^.

Furthermore, society may pressure cisgender women to have children, and if they do, it may be expected that they take time out of their careers to take care of their children^29^. Having children is associated with a significant decline in the number of publications, which may have further negative effects on success within most selection processes^30^. Furthermore, challenges faced by scientists who are mothers increase when combined with other forms of discrimination including racial and ethnic discrimination and discrimination in relation to sexual orientation^31^.

In academic evaluations, not accounting for maternity leave harms women’s productivity in the short, medium, and long terms^31^. It is worth mentioning that the societal perception of maternity leave in heterosexual couples brings a gender-biased perspective since in many cases, it is expected that the women take time off work to take care of the child, despite co-parenting responsibilities. This perception is related to the stereotyped roles in many societies of women as caregivers and men as breadwinners, where women are perceived to present greater and natural skills to take care of children, while men lack those skills and should, then, act as a secondary caregiver who only helps when needed^20^. In addition, the lack of studies focusing on the challenges faced by single-parents and 2SLGBTQIA + families prevents the development of measurements to address these challenges^32^. In any case, the current biased scenario must shift from a manager-helper dynamic towards a co-parenting perspective^20^ to positively impact family dynamics and allow women and other caregivers to spend more time on their careers and individual goals. Similarly, academic support networks for both single parents and couples must be culturally developed in the academic community to stimulate collaborations and prevent negative impacts on parent’s productivity.

Another factor contributing to the glass ceiling effect is the greater difficulty faced by women in obtaining research funding^33^. Women apply less for research funding, are more modest in the amount of money requested, less frequently awarded and receive lower amounts of funding than men, which can be an important predictor of lower academic productivity^33^. The increase in female PIs could reduce gender inequities in academia^33^. In this sense, initiatives such as L’Oreal-UNESCO Women for Science (https://www.forwomeninscience.com/) can enhance funding opportunities for women scientists.

In addition to the barriers faced in relation to productivity, potentiated by marriage and children, women are the minority in editorial boards and as journal referees^34^. Consequently, they may experience gender-biased evaluations of manuscripts, since men tend to make most review invitations to other men, besides attributing more positive reviews for papers with men as corresponding authors^18,34^. This men-men academic cycle may be associated with higher acceptance rates of papers with men as first authors or corresponding authors^18^.

This cascade of men–men positive feedback results in white men achieving higher productivity and visibility, thus being more likely to be appointed in leadership positions^15,18^. On the other hand, it also results in a biased system where female scientists receive harsher reviews, have higher rejection rates of papers, and receive fewer citations than males^34^. Thus, homophily in STEM may result in female researchers struggling to obtain mentorship, establish collaborations, achieve senior positions, and obtain research funding, decreasing women’s opportunities to hold in their career and contributing to the leaky pipeline phenomenon^2^.

Hindering effects are actual pitfalls that set back women’s long-term retention in STEM and emphasize bias in scientific environments. Increasing women’s representation in tenured positions and editorial and evaluation boards through affirmative actions would be one first step to encourage other female scientists to proceed^1,31^. Other actions to keep women in these positions include stimulating institutional support for academic mothers, establishing more diversified collaborations^31^, promoting meetings and workshops to raise awareness regarding gender and other biases in academia^1^, and creating support networks among underrepresented groups (e.g., Women in Global Health, United Nations Entity for Gender Equality and the Empowerment of Women, Organization for Women in Science for the Developing World; Kunhã Asé Network of Women in Science). Such networks are important to promote direct advocacy for women’s rights, diversity, and inclusion agendas. Furthermore, implementing inclusive success metrics for a paradigm shift in scientific values based on multidimensional mentorship promotes researchers’ well-being^24^. However, like the other initiatives, it will require collective efforts supported by academic leaders and administrators to drive essential systemic change.

## Concluding remarks

The current relative homogeneity in academia, and especially in STEM fields, is not amenable for scientific development and civil society, since diversity is associated with increased scientific innovation rates and improved solutions^35^. Thus, diversifying knowledge production and research may bring many benefits to society, allowing the contemplation of distinct perspectives^35^.

To effectively achieve equity and inclusion, not just in numbers but in real practice, we need to comprehend the multiple layers comprising our society that generate prejudice to a group of individuals, erasing their contribution to knowledge construction. Thus, understanding the gaps in the academic representation considering the concept of intersectionality is paramount to fight implicit and explicit biases and promote real progress towards a more inclusive STEM^1,3^. Actions promoting diversity, equity, and inclusion must be prioritized to stimulate democratic and diverse knowledge production.
